# Supplementary material for: Identification of photoperiod-induced specific miRNAs in the adrenal glands of Sunite sheep (Ovis aries)
Source: Front Vet Sci. 2022 Jul 22;9:888207. doi: 10.3389/fvets.2022.888207 (PMC9354845; doi:10.3389/fvets.2022.888207)
Supplement: Supplementary file 1 [file Data_Sheet_1.ZIP › Supplementary file/miRDeep2 file.zip!/miRDeep2 file/Know_Structure/SP42A2/oar-mir-148a.pdf]

miRBase precursor : oar-mir-148a
Total read count : 2213468
oar-miR-148a read count : 2210069
remaining reads : 3399

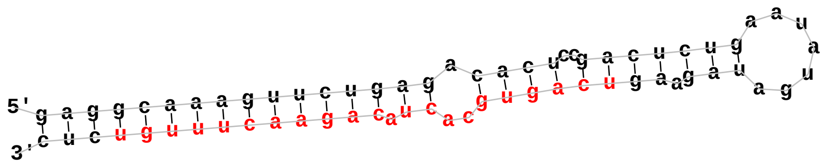

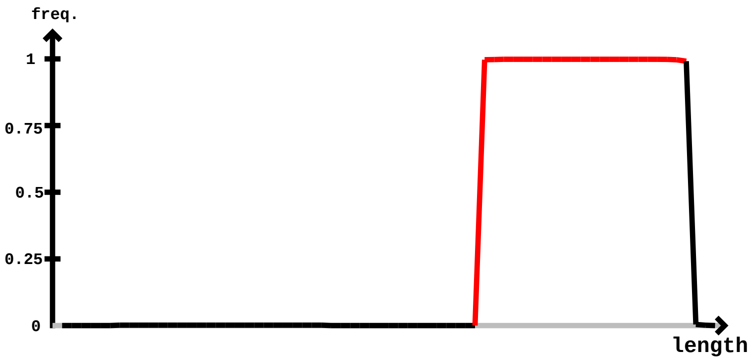

oar-miR-148a

5'- gaggcaaaguucugagacacuccgacucugaauaugauagaagucagugcacuacagaacuuugucuc -3'	exp		
((((((((((((((((.((((..(((((((.......))).))))))))..)).))))))))))))))	reads	mm	sample
...gcaaaguucugagacacuccgacu...	2	0	seq
....caaaguucugagacacuccga...	4	0	seq
....caaaguucugagacacuccgacu...	73	0	seq
.....aaaguucugagacacuc..	1	0	seq
.....aaaguucugagacacuccg..	1	0	seq
.....aaaguucugagacacuccga...	117	0	seq
.....aaaguucugagacacuccgac..	44	0	seq
.....aaaguucugagacacuccgacu...	3144	0	seq
.....aaaguucugagacacuccgacuc..	4	0	seq
.....aaaguucugagacacuccgacucu.......................................	2	0	seq
......aaguucugagacacuccgacu...	3	0	seq
...................cuccgacucugaauaugauaga...........................	1	0	seq
....................uccgacucugaauaugauaga...........................	1	0	seq
....................uccgacucugaauaugauagaag.........................	1	0	seq
....aagucagugcacuacagaacuuugu...	1	0	seq
....gucagugcacuacagaacuuug....	2	0	seq
....gucagugcacuacagaacuuugu...	77	0	seq
....guUagugcacuacagaacuuugu...	2	1	seq
....ucagugcacuacagaac........	4	0	seq
....ucagugcacuacagaacu.......	232	0	seq
....ucaCugcacuacagaacu.......	4	1	seq
....ucaguAcacuacagaacuu......	1	1	seq
....uUagugcacuacagaacuu......	3	1	seq
....ucagugcacuacagaacuu......	561	0	seq
....ucagugcacuacagaacuuu.....	3315	0	seq
....uUagugcacuacagaacuuu.....	9	1	seq
....ucagugcacuacagaacuuug....	11661	0	seq
....uUagugcacuacagaacuuug....	14	1	seq
....uUagugcacuacagaacuuugu...	3033	1	seq
....ucagugcacuacagaacuuugu...	2178455	0	seq
....ucagugcacuacagaacuuuguc..	6305	0	seq
....uUagugcacuacagaacuuuguc..	7	1	seq
....ucagugcacuacagaacuuugucu.	2881	0	seq
....ucagugcacuacagaacuuugucuc	12	0	seq
....cagugcacuacagaacuuu......	6	0	seq

oar-miR-148a

```
gaggcaaaguucugagacacuccgacucugaauaugauagaagucagugcacuacagaacuuugucuc
............................................cagugcacuacagaacuuug....     5       0       seq
............................................Uagugcacuacagaacuuugu...     9       1       seq
............................................cagugcacuacagaacuuugu...     1287    0       seq
............................................cagugcacuacagaacuuuguc..     5       0       seq
............................................cagugcacuacagaacuuugucu.     4       0       seq
.............................................aguUcacuacagaacuuu.....     1       1       seq
.............................................agugcacuacagaacuuu.....     2       0       seq
.............................................agugcacuacagaacuuug....     18      0       seq
.............................................agugcacuacagaacuuugu...     2114    0       seq
.............................................agugcacuacagaacuuuguc..     6       0       seq
.............................................agugcacuacagaacuuugucu.     6       0       seq
.............................................agugcacuacagaacuuugucuc     1       0       seq
..............................................gugcacuacagaacuuugu...     2       0       seq
...............................................ugcacuacagaacuuugu...     4       0       seq
................................................gcacuacagaacuuuUu...     1       1       seq
................................................gcacuacagaacuuugG...     1       1       seq
................................................gcacuacagaacuuugA...     1       1       seq
................................................gcacuacagaacuuugu...     18      0       seq
```
